# Comparison Between Robotic Rectopexy and Perineal Approaches in the Treatment of Rectal Prolapse: A Review of the Literature

**DOI:** 10.7759/cureus.96696

**Published:** 2025-11-12

**Authors:** Noureldine Nagui, Mohamed Abuahmed, Ben Liu

**Affiliations:** 1 Upper Gastrointestinal Surgery, The Royal Wolverhampton NHS Trust, Wolverhampton, GBR; 2 Colorectal Surgery, The Royal Wolverhampton NHS Trust, Wolverhampton, GBR; 3 General Surgery, University of Edinburgh, Edinburgh, GBR

**Keywords:** altemeier’s, delorme’s, perineal approaches, rectal prolapse, recurrent prolapse, robotic rectopexy, suture rectopexy

## Abstract

Rectal prolapse (RP) is the external protrusion of the rectum through the anus. It is either mucosal, where only the mucosal layer prolapses, or full thickness, with circumferential protrusion through the anus of all layers of the rectal wall. Surgery is the mainstay of treatment and can be performed via abdominal or perineal approaches. This literature review aimed to compare robotic and perineal approaches for treating RP in terms of outcomes, recurrence rates, and cost-effectiveness. PubMed was searched for all studies focusing on RP robotic and perineal approaches between 2015 and 2025. This timeframe was specifically selected to include as many studies incorporating robotic approaches as possible, as this is a relatively new technique. The review included nine studies on robotic approaches in comparison to 26 studies on perineal approaches. There was an obvious superiority of robotic approaches in terms of length of stay (LOS), recurrence rates, and improvement in incontinence, constipation, and quality of life (QoL) scores. Perineal approaches, on the other hand, showed better results regarding operative time and cost-effectiveness of the index procedure. It was concluded that robotic approaches for treating RP are more effective, offering shorter LOS, lower recurrence rates, and better patient satisfaction than perineal approaches.

## Introduction and background

Rectal prolapse (RP) is a condition where the rectum protrudes through the anal canal, causing significant anal pain, incontinence, and/or constipation. RP predominantly affects elderly women, with high body mass index (BMI), complications from childbirth, connective tissue disorders like Marfan syndrome and Ehlers-Danlos syndrome, which increase predisposition to RP due to weakened connective tissue integrity [1], as well as conditions like anorexia nervosa, which can lead to significant weight loss and muscle weakness, contributing to prolapse [1].

Surgical intervention is often required to restore normal anatomy and physiology. Several surgical techniques are available for RP. Literature suggests that abdominal procedures are the best curative option with a lower recurrence rate compared to perineal approaches [2]. Perineal approaches include Delorme’s procedure, which involves stripping the mucosa of the prolapsed rectum and plicating the muscular layer. It is a suitable option for elderly patients, which leads to fewer complications but higher recurrence rates [3]. Another popular perineal approach is Altemeier’s procedure, which involves resecting the prolapsed segment and performing an anastomosis. It is associated with good long-term functional outcomes, including reduced constipation and fecal incontinence and lower recurrence rates [4].

Abdominal procedures include laparoscopic ventral mesh rectopexy (LVMR), which is a widely used abdominal approach that involves attaching a mesh to the sacrum and rectum to suspend it in place. Studies have shown that LVMR is associated with fewer postoperative complications, lower recurrence rates, and improved functional outcomes compared to other techniques [5]. A second operation type is resection rectopexy, which combines rectal resection with rectopexy to address prolapse. It is often used in patients with significant constipation. Long-term recurrence rates are lower compared to ventral mesh rectopexy, particularly in primary prolapses [6].

Robotic rectopexy has emerged as a minimally invasive, promising surgical approach for treating both first-time and recurrent RP, offering improved outcomes, better correction of anatomical abnormalities, and lower recurrence rates compared to traditional methods [7]. 

The use of biological mesh in robotic rectopexy has also been safe and effective for external rectal prolapse (ERP), with significant improvements in functional outcomes and high patient satisfaction [8]. The evidence from multiple studies supports the efficacy of biological mesh in reducing symptoms such as constipation and fecal incontinence and in improving the quality of life (QoL) for patients undergoing this procedure [8]. While the use of biological mesh in robotic rectopexy shows improved results, it is important to consider the limitations of the current evidence. Most studies are retrospective or observational, and there is a lack of randomized controlled trials directly comparing different types of biological meshes or their long-term outcomes [9]. This study aimed to offer a review of what the current literature recommends regarding the operative management of RP and functional outcomes and complications of both perineal and robotic approaches.

Methodology

This literature review provides an overview of all the studies conducted on robotic rectopexy, with or without mesh, in comparison to perineal procedures for the repair of RP over the past 10 years, specifically between 2015 and 2025, as published in PubMed. The Boolean search terms used were: robotic mesh rectopexy AND robotic suture rectopexy AND perineal approach OR Delorme’s procedure OR Altemeier’s procedure AND rectal prolapse. Additionally, this review included observational studies, randomized controlled trials, as well as systematic reviews with meta-analysis comparing robotic abdominal procedures to perineal procedures for the treatment of first-time and recurrent RP. The data included age, gender, presenting symptoms, operative time, length of stay (LOS), postoperative complications, conversion to open rates, recurrence rates, improvement in incontinence scores, improvement in constipation scores, and improvement in QoL scores. Lastly, median follow-up period and cost-effectiveness studies were also reviewed and used to evaluate results. Exclusion criteria comprised studies focusing on laparoscopic approaches, index open abdominal approaches, as well as stapled transanal rectal resection (STARR).

Statistical analysis was conducted using Jamovi software v2.7.6 (Jamovi Project, Sydney, Australia; www.jamovi.org). Descriptive data were presented as mean and standard deviation, whereas quantitative variables were analyzed using the Student's t-test for two variables and one-way analysis of variance (ANOVA) for three or more variables. Qualitative variables were analyzed using the chi-square test.

No ethical concerns were raised in this study, as it did not include any direct involvement with patients and was purely a review of the literature.

## Review

RP, especially recurrent RP, is a challenging condition that significantly impacts patients' QoL. Surgical interventions are the primary treatment, but the optimal approach remains debated. Abdominal rectopexy generally carries lower recurrence rates (5.3%-10.8%), improved functional outcomes, and longer hospital stay, while the perineal approach has higher recurrence rates (16.3%-38%), shorter and fewer hospital stays, but similar recurrence rates to laparoscopic surgery. The latter approach itself has comparable functional outcomes to the robotic approach, but with a longer LOS and is more cost-effective [10]. Moreover, the use of mesh rectopexy carries lower recurrence rates (4.1%-7.7%) than suture rectopexy, but with longer operative duration than the latter, and has rarely reported mesh complications [11].

We reviewed the literature for all studies conducted in the last 10 years, comparing robotic suture rectopexy, robotic mesh rectopexy, Delorme’s procedure, and Altemeier’s procedure for both first-time and recurrent RP. More than 80 studies were reviewed, and 26 studies in total were found eligible for this review. The studies on robotic rectopexy included 348 patients, compared to 6,828 patients who underwent perineal repair of RP. Both approaches were compared in terms of operative time, LOS, recurrence rates, 30-day complication rates, functional outcomes, and the duration of follow-up. Table 1 presents a comparison between these studies, highlighting complications, improvement of functional status, and recurrence rates.

**Table 1:** Comparison between studies addressing robotic rectopexy approaches and perineal approaches. * Chi-square test was used to analyze the difference in morbidity rates. ** Student's t-test was used to analyze the difference in mean operative time. *** Student's t-test was used to analyze the difference in mean LOS. † Chi-square test was used to analyze the difference in recurrence rates. ‡ ANOVA test was used to analyze the difference in mean improvement in incontinence scores. § Student's t-test was used to analyze the difference in follow-up months. ANOVA: analysis of variance; LOS: length of stay; IQR: interquartile range; NA: not applicable

Parameter	Robotic rectopexy approach	Perineal approach			Test value
	Mesh rectopexy	Total	Delorme's procedure	Altemeier's procedure	
Number of studies	9	26	13	13	NA
Total number of patients	348	6828	5655	1173	NA
Morbidity (%) *	27 (7.7%)	556 (8%)	495 (8.7%)	61 (5.2%)	χ^2^: 0.065 (p=0.79)
Operative time in minutes (mean) **	143.5 (±55.5)	82 (±25)	83.9 (±27.4)	83.1 (±28.33)	t: 35.517 (p<0.0001)
LOS in days (mean) ***	3.4 (±13)	6.9 (±3.7)	6 (±5.4)	6 (±5.5)	t: 13.43 (p<0.0001)
Recurrence (%) †	8 (2.29%)	415 (6%)	278 (4.9%)	137 (11.6%)	χ^2^: 0.065 (p=0.0035)
Improvement in incontinence score (mean) ‡	17.9 (±18.18)	14.2 (±14.8)	16.3 (±20.3)	6.8 (±8.7)	F-statistic: 0.10266 (p=0.9)
Follow-up in months (median/IQR) §	24 (56)	36 (55)	41 (53.78)	35 (56)	t: 3.966 (p<0.0001)

Recurrence rates

The findings regarding recurrence rates in RP surgery reveal a complex variation, particularly when comparing robotic rectopexy to perineal methods. Existing literature generally indicates that abdominal approaches result in lower recurrence rates than perineal techniques [12-14]. Nevertheless, a recent systematic review and meta-analysis including 15 studies and 1,611 patients found that non-randomized controlled studies reported a lower risk of recurrence with the abdominal approach than with the perineal approach (odds ratio (OR) 0.29, 95% confidence interval (CI): 0.17 to 0.50; p<0.00), while in randomized controlled trials, no difference was observed (p=0.720) in the risk of recurrence with the abdominal approach compared to the perineal approach (9.8% vs. 16.3% (OR 0.82, 95% CI: 0.29 to 2.37; p=0.720)) [12]. Our current review supports these findings, showing that robotic rectopexy exhibited a recurrence rate of 2.29% compared to 6% for perineal approaches - a difference that was statistically significant (χ^2^=0.065, p=0.0035). However, this difference might be affected by the variation in follow-up period between the two groups, with the median follow-up of 24 months in the robotic group compared to 36 months in the perineal group (t=3.9667, 95% CI: -17.94 to -6.06, p<0.0001).

LOS

The abdominal approach is associated with longer hospital stays compared to the perineal approach. Studies have reported an average LOS of seven to 10 days for abdominal procedures compared to four to seven days in the case of perineal procedures [12,13,15]. A systematic review with meta-analysis highlighted that the median LOS for perineal approaches was 4.9 days, significantly shorter than the 7.2 days for abdominal approaches [13]. These findings were reinforced by data collected from a randomized trial including 134 patients conducted in Sweden, where the LOS in the perineal group was significantly lower than the abdominal group (p=0.022) [16]. However, in our review, the calculated mean LOS in the case of robotic rectopexy was 3.4 (±13) days compared to 6.9 (±3.7) days in the perineal group (t=13.4364, 95% CI: -3.897 to -2.903, p<0.0001) [9,17,18].

Operative duration

Robotic rectopexy tends to have a longer operative duration compared to perineal methods [17]. This is due to the complexity and precision required in robotic techniques, especially in the case of mesh rectopexy, which has been shown to have a longer mean operative time compared to laparoscopic rectopexy due to a longer learning curve [19].

On the other hand, perineal procedures like Altemeier’s and Delorme’s are generally quicker to perform. Furthermore, a systematic review comparing these two perineal techniques found no significant difference in operative time between them, indicating that perineal approaches are relatively time-efficient [20]. This information is highlighted in our review, which found that the mean operative duration was 143.5 (±55.5) minutes in robotic rectopexy compared to 82 (±25) minutes in the perineal approach (t=35.517, 95% CI: 50.824 to 56.776, p<0.0001). This was also emphasized by a randomized controlled trial that compared the abdominal and perineal approaches, where the mean operative duration was 139 (±82) minutes for abdominal approach in comparison to 76 (±35) minutes for perineal approach; however, the results were not statistically significant (p=0.061) and clear distinction between robotic and laparoscopic approaches was not clearly made in that study [16].

Thirty-day postoperative complications

While abdominal procedures are associated with better functional outcomes and lower rates of recurrence, they may have a higher risk of certain complications. A prospective study conducted in 2024 included 149 patients and showed that patients who underwent robotic-assisted RP repair had a 10.4% rate of minor complications (Clavien-Dindo grade I-II). Three patients had urinary retention, requiring a one-time recatheterization. Four patients developed a urinary tract infection and were treated successfully with medical therapy [21]. On the other hand, perineal approaches are generally considered safer with lower immediate postoperative morbidity, which makes them a better option for high-risk patients due to their less invasive nature. When comparing both approaches, Altemeier’s procedure, for example, has shown better outcomes in terms of lower anastomotic leak rates compared to Delorme’s procedure [20]. In our review, robotic rectopexy procedures resulted in 27 patients having 30-day morbidity (7.7%). On the other hand, the perineal approach resulted in a total of 556 patients (8%) having complications in the first 30 days postoperatively, with there being a trivial difference between both Delorme’s and Altemeier’s procedures. This difference between robotic and perineal approaches was not statistically significant (χ^2^=0.065, p=0.79).

Functional outcomes

Current literature demonstrates that abdominal approaches may result in higher postoperative constipation rates but improved incontinence scores [12,13], while perineal approaches are associated with better correction of constipation but may worsen incontinence in some cases [22]. In our review, the included studies showed an improvement in incontinence scores following robotic mesh rectopexy (mean 17.9±18.18), Delorme’s procedure (mean 16.3±20.3), and Altemeier’s procedure (mean 6.8±8.7). However, these differences were not statistically significant (F-statistic: 0.10266, p=0.9). Regarding improvement in constipation, an average of 19.5% improvement in constipation score was observed among studies including mesh rectopexy patients. Additionally, one study demonstrated an improvement in constipation postoperatively in 87% of the patients [18]. On the other hand, one study included in the review reported a better constipation score, with an average improvement of 4.2% [23], while two studies showed better postoperative constipation in an average of 49.8% of the patients [22,24].

Patient satisfaction and QoL

Patient satisfaction and QoL after robotic rectopexy are generally high. A study reported that 62% of patients who underwent robotic ventral rectopexy had low postoperative constipation, incontinence, and pain, high sexual function scores, and significant improvement in their overall condition [25]. Another study found that the subjective satisfaction rate was 87% in the robotic ventral mesh rectopexy group, although this difference was not statistically significant compared to the laparoscopic group [17]. The same study found that the changes in health-related quality of life (HRQoL) were remarkably improved in the robotic group at three months, although these improvements declined in both groups at two years.

In our review, a randomized controlled trial that compared robotic with laparoscopic mesh rectopexy showed an overall improvement in QoL scores, with mean improvement in Colorectal-Anal Impact Questionnaire (CAIQ-7) of -25 (±30.1), Pelvic Organ Prolapse Quantification (POPIQ-7) of -2.5 (±30.3), Urinary Impact Questionnaire (UIQ-7) of -2.9 (±39.4), Pelvic Floor Impact Questionnaire (PFIQ-7) of -30.2 (±89), and 15-Dimensional Health-Related Quality of Life (15D) score of 0.011 (±0.12) in the robotic group [17]. On the other hand, another randomized controlled trial compared Delorme’s procedure to laparoscopic mesh rectopexy and showed a statistically significant difference in Gastrointestinal Quality of Life Index (GIQLI) score (p<0.0001) [24]. 

Cost-effectiveness 

The cost-effectiveness of abdominal versus perineal approaches is influenced by the balance between upfront costs perioperatively and long-term outcomes. Abdominal procedures, despite higher initial costs, may be more cost-effective in the long run due to lower recurrence rates and better functional outcomes [26]. In our review, three studies demonstrated the cost of robotic rectopexy with an average of £4850 [17,21,27]. While specific cost data for the perineal approach are not clear in the current literature, factors like operative time, hospital stay, and complication rates are key determinants of the overall cost. There is no official data from the National Health Service (NHS) that demonstrates the actual costs of Delorme’s or Altemeier’s procedure. However, costs are estimated between £2000 and £4000 based on average theatre costs and LOS [28].

Conversion to open surgery rates

Conversion to open surgery is rare for both robotic and laparoscopic approaches, with rates ranging from 0% to 3% [17,19]. The learning curve for robotic methods may slightly increase conversion rates in the early stages, but this improves with surgeon experience. In this review, only two studies reported conversion to open surgery from robotic mesh rectopexy. One of them reported that only one patient (2.9%) had a conversion to open surgery due to a small enterotomy detected during robotic surgery. As a result, a small midline laparotomy was performed to repair the small bowel [10]. The other study showed one patient as well (4%) who had a conversion to open surgery due to extensive pelvic adhesions [29].

Recurrent RP

We reviewed the literature for the management of recurrent RP, as it represents an important entity due to the complexity of cases sometimes. Five studies included patients with recurrent RP following both perineal (204 patients) and abdominal (77 patients) approaches. Among these, 118 patients had their recurrent prolapse repaired by robotic mesh rectopexy, 102 patients by Delorme’s procedure, and 50 patients by Altemeier’s procedure [6,12,30-32].

Regarding complication rates, only two patients (1.6%) who underwent the robotic approach for recurrent prolapse experienced postoperative complications compared to 17 patients (11%) after the perineal approach [31,33]. In addition, 24 patients (20%) developed a second recurrence of prolapse following robotic repair compared to 33 patients (21.7%) after perineal approach [30,31]. Only one patient had conversion to laparoscopic surgery (3.2%) due to extensive pelvic adhesions [29].

Regarding functional outcomes, there was a significant improvement in incontinence and constipation scores by 21.75% and 22.75%, respectively, with a mean follow-up period of 67 (±42.8) months [6,8].

Limitations of this study include the discrepancy in the number of patients between the robotic group and the perineal group; this might be attributed to the lower number of studies conducted on robotic RP repair. Another limitation is the scarcity of randomized trials of high quality in this review, which might have affected the results. Moreover, there is an underrepresentation of robotic suture rectopexy approach except for some sporadic case reports, which means that further observational and randomized controlled trials are required to compare robotic mesh rectopexy with suture rectopexy. Finally, no comparison could be made between robotic and perineal approaches regarding cost-effectiveness due to a lack of detailed studies addressing the costs of perineal techniques such as Delorme’s or Altemeier’s.

## Conclusions

Robotic rectopexy, with or without mesh, offers a more effective approach with shorter LOS and lower recurrence rates than perineal approaches. The robotic technique is also superior in terms of QoL, constipation, and incontinence scores. Howver, operative duration remains a major drawback of the robotic approach. It is expected to improve in the future due to the better learning curve of robotic surgeons. Although the cost of the index robotic procedure is higher than that of perineal approaches, the fact that perineal approaches lead to more recurrence rates, more outpatient hospital visits, and recurrent surgeries compensates for this cost difference.
